# Alcoholic extracts of *Teucrium polium* exhibit remarkable anti-inflammatory activity: In vivo study

**DOI:** 10.17305/bb.2023.9239

**Published:** 2024-02-01

**Authors:** Hamda A Al-Naemi, Reem Moath Alasmar, Kaltham Al-Ghanim

**Affiliations:** 1 Laboratory Animal Research Centre, Qatar University, Doha, Qatar; 2 Department of Biological and Environmental Sciences, Qatar University, Doha, Qatar; 3 Social and Economic Survey Research Institute (SESRI), Qatar University, Doha, Qatar

**Keywords:** Ethanolic plant extract, acute inflammation, α-carrageenan, paw edema.

## Abstract

*Teucrium polium* (germander, Lamiaceae) is a local plant in Qatar that has been used in folk medicine to treat numerous illnesses. It is known for its antioxidant, analgesic, anticancer, and antibacterial activities. This study aimed to evaluate the anti-inflammatory activity of *Teucrium polium* (TP) extract by α-carrageen-induced paw edema in adult Sprague-Dawley rats. The animals were randomly grouped into control, acute inflammation, and plant extract groups*.* Acute inflammation was induced by a sub-plantar injection of 100 µL of 1% α-carrageenan into the rat’s right hind paw. Three different doses of the ethanolic extract of TP were tested at different time periods (1, 3, and 5 h). All doses of the TP ethanolic extract showed significant inhibition of α-carrageenan-induced rat paw edema in a dose-dependent manner in both early and late phases of edema formation. The size of the α-carrageen-induced paw edema was significantly reduced one, three, and five hours after TP extract injection compared to the acute inflammation group. This inhibition was accompanied by high expression of interleukin 10 (IL-10) and low expression of monocyte chemoattractant protein 1 (MCP-1), interleukin 1 beta (IL-1β), and tumor necrosis factor alpha (TNF-α). The results indicated that the ethanolic extracts of TP possess significant anti-inflammatory and potential pharmaceutical properties.

## Introduction

Inflammation is a controlled physiological response that occurs after tissue injury by physical, chemical, and biological agents. It is characterized by different biological and immunological events [1]. Uncontrolled inflammation can trigger pathophysiological and immunological events that might result in pathological conditions, such as allergies, irritable bowel syndrome, and cancer [2]. The inflammatory response can be either acute or chronic. The acute phase is an initial stage of the reaction characterized by white blood cells infiltration, high vascular permeability, and extravasation of plasma causing tissue edema [1]. It lasts for an hour to five hours and is characterized by the activation of neutrophils and macrophages as well as the release of pre-synthetized inflammatory mediators, such as histamine and eicosanoids. Activated neutrophils produce pro-inflammatory cytokines, such as interleukin 1 beta (IL-1β), IL-6, and tumor necrosis factor alpha (TNF-α) [3, 4]. Acute inflammation can be induced by α-carrageenan to evaluate the anti-inflammatory activity in animal models under experimental conditions [5, 6]. The formation of tissue edema in the paw results from a synergistic interaction between different inflammatory mediators that increase vascular permeability and/or increase blood flow and can be resolved within five hours [4]. If not resolved, inflammation provokes the development of chronic inflammatory responses and disorders, such as chronic inflammatory skin diseases, asthma, arthritis, autoimmune diseases, and inflammatory bowel diseases [4, 7]. In some cases, these conditions can be fatal if not properly managed and regulated. The most common drugs used to treat inflammation are nonsteroidal anti-inflammatory drugs (NSAIDs) and steroidal anti-inflammatory compounds [8]. However, NSAIDs cannot be used in patients with gastric ulcers, high blood pressure, kidney damage, and cardiovascular disease due to their multiple side effects. Therefore, there is a need to evaluate and explore new natural molecules with anti-inflammatory activity and potential therapeutic use. In recent years, research has been oriented toward developing anti-cytokine agents from herbal extracts based on their effectiveness in controlling various inflammatory diseases [9, 10].

Traditional healers in different countries in Africa and Asia use a wide range of traditional medicinal plants [11]. *Teucrium polium* (TP) is a subshrub and a seasonal plant distributed mainly in arid and semiarid regions of North Africa, the Mediterranean region, and Southwest Asia. It belongs to Phylum: Tracheophyta, Class: Magnoliopsida, Order: Lamiales, Family: Lamiaceae, Genus: Teucrium L., Species: Teucrium polium L. Aerial parts of TP are used in traditional medicine to treat stomach and intestinal disorders. TP is an endemic plant in Qatar, locally known as “Jaada” [12]. Traditionally, the aerial parts of TP are boiled and used to treat fever, constipation, and as an antispasmodic agent [13].

**Figure 1.**
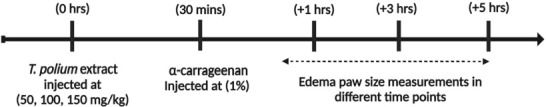
**Experimental timeline**. Rats were injected in different time periods with *T. polium* extract and then with α-carrageenan to induce the inflammation. Paw edema size was measured after 1, 3, and 5 h.

Phytochemical analysis of the aerial parts revealed the presence of potentially bioactive compounds, such as flavonoids and terpenoids [13]. Such compounds are well known for their pharmacological effects, such as hypoglycemic, anti-inflammatory, hepatoprotective, antifungal, and antibacterial activities [14, 15]. Results from in vitro study showed that the crude ethanolic extract contains phenolic and flavonoid compounds with anti-inflammatory activity [16, 17]. It has been reported that TP extracts have antioxidant, antibacterial, and anti-inflammatory activities that can be explored for potential therapeutic uses. Recently, it has been reported that pregnant women could be very sensitive to these kinds of medical plants, especially in the early stage of pregnancy [18]. Therefore, the aim of the present study is to investigate the anti-inflammatory activity of TP extract to confirm its effectiveness in animal models.

## Materials and methods

### Drugs and reagents

Acute inflammation was induced by α-carrageenan (Sigma chemical CAS Number: 9000-07-1, Co., St. Louis, U.S.A). Clinical grade meloxicam (Intracin Pharmaceutical Pvt. Ltd., Batch No. 201173-GUJARAT, India) was used as an anti-inflammatory reference drug.

### Collection and identification of plant crude extract

The fresh aerial parts of TP (stems, leaves, and flowers) were collected by Dr. Kaltham Al-Ghanim (Qatar University, Doha, Qatar) in April 2022 in the northern region of Qatar. The plant material was authenticated and taxonomically identified at the Qatar University Herbarium - Department of Biological and Environmental Sciences. The aerial parts of the plants were dried in the shade at a room temperature of 25 ^∘^C for two weeks. After the drying process was complete, the plants were stored in the fridge at 4 ^∘^C in clean containers for further analysis.

### Preparation of the crude extract

The aerial plant parts of TP were mechanically ground to a fine powder using a grinder and 20 g of the plant material was macerated in 80% ethanol and placed in a water bath at 45 ^∘^C for 72 h with continuous gentle shaking at 45 ^∘^C. Then the solvent extract was filtered with filter paper (Whatman No: 1) and evaporated from the ethanol in the oven (Thermo Scientific™ Heratherm™ ) at 45 ^∘^C. The remaining residue crude extract was conserved in glass containers at 4 ^∘^C [19]. The residual crude extract was dissolved in 0.9% saline water and filtered through a 0.25-µm syringe filter. The drug was prepared based on the three-dose concentration (50, 100, 150 mg/kg B.wt).

### Experimental design

Adult male Sprague-Dawley rats (12 weeks old) were obtained from Qatar University Laboratory Animal Research Center (LARC) breeding colony and kept at LARC in individually ventilated cages under controlled environment of 22 ± 2 ^∘^C, 60% relative humidity, and a cycle of 12 h light and 12 h dark. All animals were provided with a standard chow diet and water ad libitum. A total of 108 animals were randomly assigned to the following groups: Group I (G-I) was the control group in which the rats received an intraperitoneal (IP) dose of 0.9% saline. Group II (G-II) was the acute inflammation group in which paw edema was induced by subplantar injection of a dose of α-carrageenan (1%) to the right hind paw of rats. Group III (G-III) was the reference control in which rats received a dose of meloxicam (4 mg/kg B.wt, IP). Group IV (G-IV) was the ethanolic plant extract-treated group subdivided into three groups, in which the rats received a single dose of different concentrations of TP extract dissolved in 0.9% saline (50, 100, 150 mg/kg B.wt) 30 min before α-carrageenan injection (Figure 1). The doses were selected based on the reports of Amraei et al. [20].

### Evaluation of anti-inflammatory activity

The anti-inflammatory activity of TP extract was evaluated by modified methods of the α-carrageenan-induced rat paw edema model [21]. Acute inflammation was induced by subplantar injection of 100 µL of 1% α-carrageenan (1% suspension in saline) into the right hind paw of the rat. The size of the edema on the rat paw was measured with a Digital Vernier caliper before and after injection of TP extract, meloxicam, and α-carrageenan at different time points (1, 3, 5 h). The increase in the diameter of the paw size was used as an indicator of edema. Edema was calculated using the following equation [22]:

(1) $$(\%)Edemainhibition=\left(T_{0}-\frac{T_{t}}{T_{0}}\right)\times 100$$

T_t_ is the paw edema size at different times and T_0_ is the paw edema size before inflammation induction by α-carrageenan.

### Tissue extraction and cytokine measurements using ELISA

Hind paws tissue was dissected above the ankle joint, snap-frozen in liquid nitrogen, and stored at −80 ^∘^C for further analysis. A piece of paw tissue (10 mg) was extracted from each sample and placed in 4 mL tissue extraction buffer provided with Raybio Rat Enzyme-linked immunosorbent assay kit. Each sample was then thoroughly homogenized using a mechanical tissue homogenizer. Samples were centrifuged at full speed for 15 min, the supernatant was extracted, and the levels of TNF-α and IL-1β were determined.

**Figure 2.**
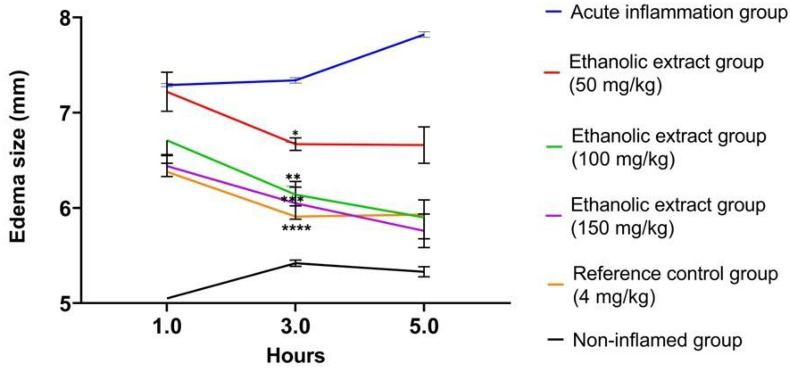
**Anti-inflammatory activity of ethanolic extract of *T. polium* plant on paw edema size.** Change in inflamed paw size treated by ethanolic plant extract doses (50, 100, 150 mg/kg), and reference control (4 mg/kg B.wt) groups after induced inflammation by α-carrageenan. Results are expressed as the mean ± SED (*n* ═ 6) (*P< 0.05, **P< 0.01, ***P< 0.001 ****P< 0.0001).

### Quantitative real-time PCR

Total mRNA was extracted using TRIzol reagent (Invitrogen). Edema paw tissue was collected, homogenized in the presence of Trizol, and stored at −80 ^∘^C. Total mRNA was extracted from the frozen tissue samples as described in the manufacturer’s protocol. The concentration and quality of mRNA were measured using nanophotometer (IMPLEN, GE Healthcare, UK). A known quantity of mRNA was transformed to first strand complementary DNA (cDNA) by incubation with Reverse Transcriptase using Kit (Applied Biosystem) using Proflex PCR machine (Applied Biosystem). Gene expression analysis was carried out using specific Taq-man primers for each inflammatory marker (IL-1β, IL-10, IL-6, TNF-α, monocyte chemoattractant protein 1 [MCP-1], and nuclear factor kappa B [NF-κB]) and analyzed using the Quantstudio 6 Flex Real-Time PCR system (Applied Biosystem).

### Ethical statement

The animal protocol was reviewed and approved by Qatar University Institutional Animal Care and Use Committee (IACUC) no: 1719825-1.

### Statistical analysis

Results are expressed as mean ± standard error deviations (SED). Statistical variation among groups was conducted by ANOVA and Prism software (version 9.4 for Windows), followed by Duncan’s test to compare the variations among groups. Differences were considered statistically significant at *P* value < 0.05.

## Results

### α-carrageenan-induced rat paw edema

**Figure 3.**
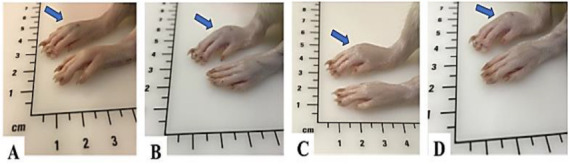
**Anti-inflammatory activity determined by an increase in right hind paw edema size in different experimental groups at the third hour after induced inflammation.** (A) Control: Non-inflamed; (B) Acute inflammation: α-carrageenan-induced inflammation; (C) Ethanolic extract group: *T. polium* treatment at a dose of 150 mg/kg B.wt + α-carrageenan-induced inflammation; (D) Reference control group: Meloxicam at a dose of 4 mg/kg B.wt + α-carrageenan.

The anti-inflammatory effect of TP ethanolic extracts was evaluated by α-carrageen-induced paw edema. The size of inflamed paw edema was reduced at 1, 3, and 5 h after TP extract injection compared with the acute inflammation group (G-II) (Figures 2 and 3). The dose concentration of 50 mg/kg B.wt resulted in a reduction of 6.56 ± 0.16 mm in the edema size at the 3rd hour of the inflammatory process, while a dose concentration of 100 mg/kg B.wt resulted in a reduction of 6.14 ± 0.29 mm in the edema size and the dose concentration of 150 mg/kg B.wt of TP extract resulted in a reduction of 6.05 ± 0.41 mm in the edema size (Figure 2).

### Evaluation of the anti-inflammatory activity of TP extracts

The percentage inhibition was dose-dependent and produced anti-inflammatory effects at different time periods (1, 3, and 5 h). Pretreatment with TP extract or the reference drug (meloxicam) had a significant percentage inhibition of inflammation (54.72% vs 34.71%) at the 1st hour, but it had a high percentage of inhibition (67.98% vs 50.19%) and (83.64% vs 59.74%) at the 3rd and 5th hours, respectively, at a dose of 150 mg/kg (*P* > 0.05). It was found that the percentage inhibitory effect at these time points increased in a dose-dependent manner.

**Figure 4.**
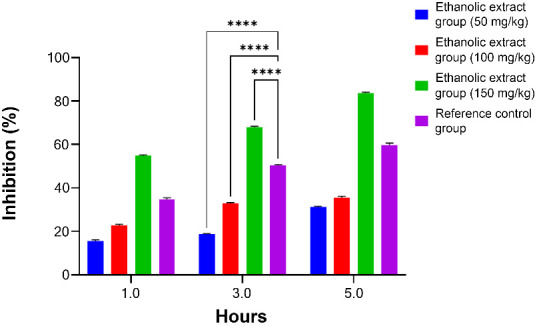
**Percent inhibition of edema in Sprague-Dawley rats treated with ethanolic crude extract of *T. polium*.** Shows change in inflammation level determined as inhibition (%) in ethanolic plant extract group (50, 100, 150 mg/kg B.wt), and reference group (4 mg/kg B.wt) after induced inflammation by α-carrageenan. The inhibition data are expressed as mean ± SEM (*n* ═ 6) (*P< 0.05, **P< 0.01, ****P< 0.001) compared to the reference drug (meloxicam) group.

The high dose (150 mg/kg B.wt) of TP extract resulted in the highest percentage inhibition of inflammation in paw edema, specifically at the peak of the inflammatory process at the 3rd hour, in contrast to the medium dose (100 mg/kg B.wt) and the lower dose (50 mg/kg B.wt) compared with the reference drug group (G-III) (Figure 4). 

### Effect of TP extracts on pro-inflammatory TNF-α and IL-1β cytokines production

**Figure 5.**
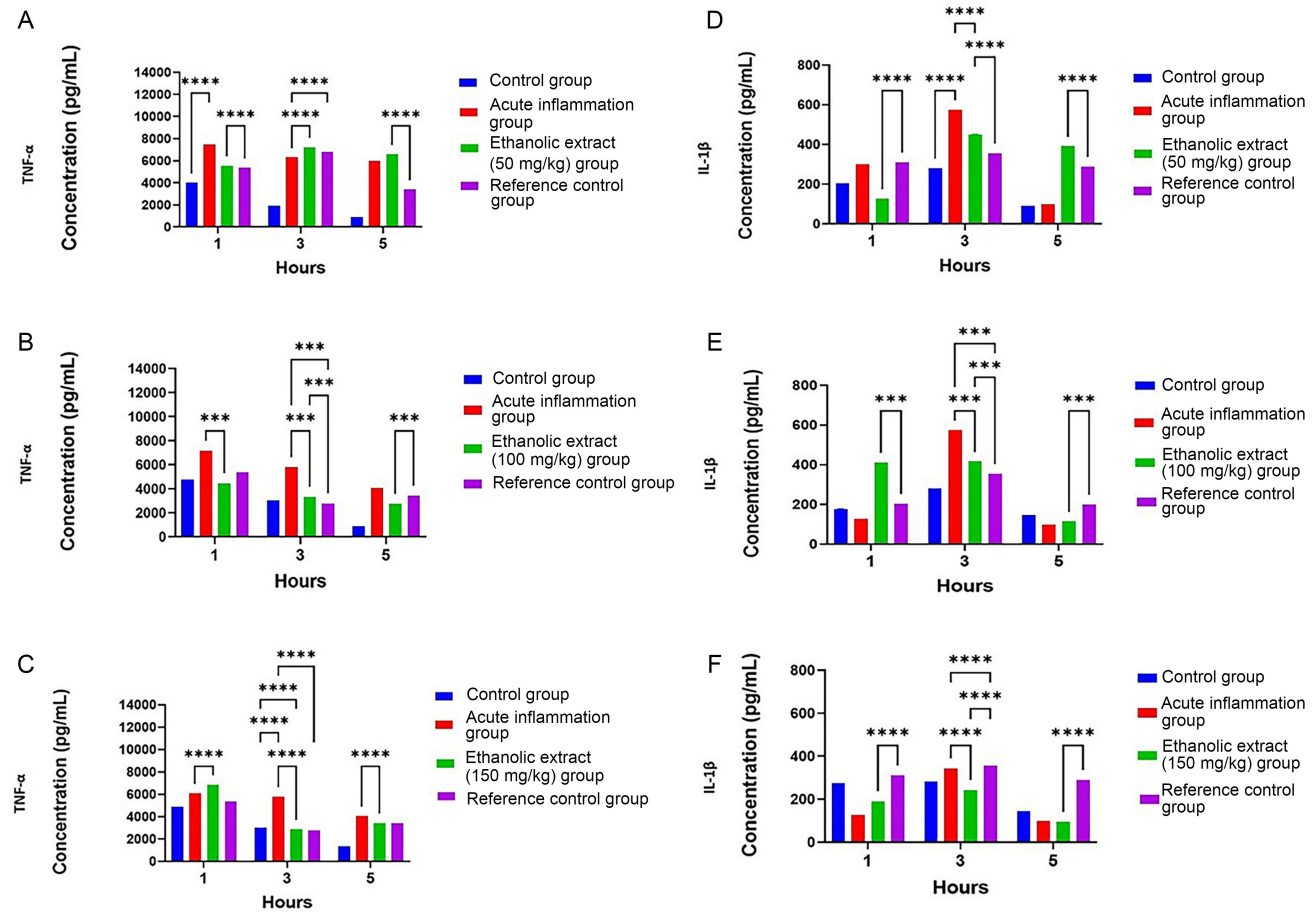
**Effect of *T. polium* ethanolic extract at different doses.** (A) Ethanolic extract at a dose 50 mg/kg, (B) 100 mg/kg, and (C) 150 mg/kg on level of TNF-α and (D) ethanolic extract at dose 50 mg/kg, (E) 100 mg/kg, and (F) 150 mg/kg on level of IL-1β after 1, 3, and 5 h from α-carrageen-induced paw edema in Sprague-Dawley male rats. Data are shown as mean ± SEM, (*n* ═ 6) (*P< 0.05, **P< 0.01, ***P < 0.001, ****P < 0.0001). IL: Interleukin; TNF-α: Tumor necrosis factor alpha.

ELISA showed that TP extract had inhibitory effect on proinflammatory cytokines (TNF-α and IL-1β) levels compared with α-carrageen-induced acute inflammation group (G-II) (Figure 5). Ethanolic TP extract significantly reduced mean level of TNF-α production at concentration of 50 mg/kg (7210.1 pg/mL ± 0.025), 100 mg/kg (3332.25 pg/mL ± 0.018), and 150 mg/kg (2816.32 pg/mL ± 0.027). Also, the extract had a significant reduction in the mean level of IL-1β at a concentration of 50 mg/kg (450 pg/mL ± 0.019), 100 mg/kg (416.446 pg/mL ± 0.004), and 150 mg/kg (250 pg/mL ± 0.002). The results WERE consistent with percentage inhibition of edema size results (Figure 5).

### Effects of TP extract on pro-inflammatory IL-1β, IL-10, IL-6, TNF-α, MCP-1, and NF-kB cytokine expression

**Figure 6.**
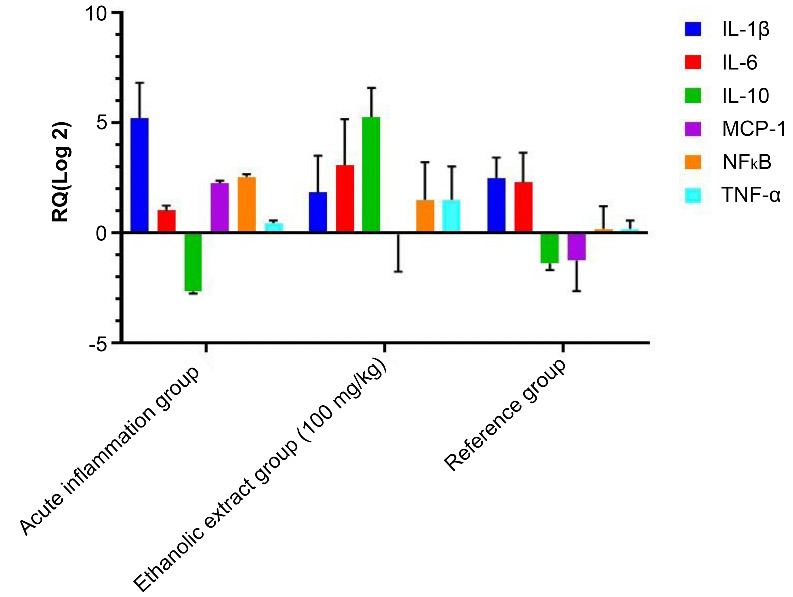
**Gene expression analysis of inflammatory markers in paw tissue samples.** The expression of IL-1β, IL-6, IL-10, TNF-α, MCP-1, and NF-κB mRNA was measured by real-time PCR in acute inflammation group (1% α-carrageenan), in ethanolic extract of *T. polium* at dose 100 mg/kg B.wt at 3 h, and reference group (4 mg/kg B.wt) at 3 h. IL: Interleukin; TNF-α: Tumor necrosis factor alpha; MCP-1: Monocyte chemoattractant protein 1; NF-κB: Nuclear factor kappa B.

The qPCR analysis of inflammatory markers (IL-1β, IL-10, IL-6, TNF-α, MCP-1, and NF-κB) showed that the expression of these inflammatory markers was suppressed by TP ethanolic extract at a dose of 100 mg/kg at the 3rd hour (Figure 6).

## Discussion

Phytochemical screening of the TP crude extract showed the presence of flavonoids, phenolics, and alkaloid compounds. These bioactive compounds make TP a potent candidate for potential pharmaceutical use to promote human health [8, 23, 24]. Several studies have demonstrated that flavonoids have anti-inflammatory properties in acute and chronic inflammation [8, 25]. However, precaution should be taken with the use of TP for prolonged periods of time. High doses (200 mg/kg) of TP for 28 days have been reported to exhibit toxic effects on different organs, such as the kidney and liver [24]. The effects of the crude extract of TP cannot be attributed to a single phytochemical moiety [8, 23]. Many studies attributed anti-inflammatory properties of TP to the presence of flavonoids [15, 26].

In the present study, the anti-inflammatory activity of the ethanolic extract of TP was evaluated in an experimental animal model of acute inflammation. The effect of TP extract on some signaling pathways involved in the induction/inhibition of acute inflammation in the α-carrageenan-induced rat paw edema was evaluated. This method is accepted for testing the biphasic events of acute inflammation [25]. The first phase (1–3 h) is mediated by the release of histamine and serotonin, the increase of vascular permeability and the formation of edema [23]. The second phase (3–5 h) is mediated by the release of pro-inflammatory cytokines and prostaglandins [8]. Our data showed that TP ethanolic extract has a potent anti-inflammatory activity that reduced the size of α-carrageenan-induced edema in a dose-dependent manner and decreased the level of acute inflammatory mediators (IL-1β and TNF-α) after three hours (Figure 2).

One possible reason for the anti-inflammatory activity of the extract in the first phase could be because of the suppression of histamine signaling. The TP could have a stabilizing effect on mast cells and a direct inhibition of the histamine H1 receptors [23]. In contrast, the anti-inflammatory activity of the TP extract in the second phase of the study (3–5 h) might be related to the anti-inflammatory effect of IL-10. Our data demonstrated for the first time an upregulation of IL-10 expression by treatment with TP. It is well documented that IL-10 has an anti-inflammatory effect. IL-10 can inhibit the synthesis of pro-inflammatory mediators: IL-β1, TNF-α, and MCP-1 [27]. This study showed that the alcoholic extract of TP upregulated the gene expression of the anti-inflammatory cytokine IL-10 and downregulated the gene expression of MCP-1, whereas both IL-10 and MCP-1 were downregulated by meloxicam treatment.

The high expression of IL-10 probably mediated the inhibition of MCP-1 and the resulting control of migration and infiltration of monocyte/macrophages and the production of pro-inflammatory cytokines (IL-β1, IL-6, and TNF-α) [28]. On the other hand, meloxicam exerts its action through the inhibition of prostaglandin synthase and decreases the production of prostaglandins [29, 30]. These results showed that the ethanolic crude extract of TP has bioactive components with anti-inflammatory properties that could suppress both phases of acute inflammation through activation of internal inhibitory mechanisms. These data could have great pharmaceutical benefits for the development of a new anti-inflammatory drug.

## Conclusion

Ethanolic extracts of *T. polium* possess potent anti-inflammatory properties, which are likely mediated by increasing the expression of anti-inflammatory cytokines IL-10, decreasing the expression of MCP-1 and decreasing the levels of pro-inflammatory mediators (IL-1β, IL-6, and α-TNF). These findings suggest that TP ethanolic extract has anti-inflammatory properties that should be explored for potential pharmaceutical applications.
